# Rethinking Metabolic Dysfunction in Chronic Liver Diseases

**DOI:** 10.17925/EE.2026.22.1.5

**Published:** 2026-02-26

**Authors:** Lung-Yi Loey Mak

**Affiliations:** 1. Department of Medicine, School of Clinical Medicine, The Li Ka Shing Faculty of Medicine, The University of Hong Kong, Hong Kong, China; 2. State Key Laboratory of Liver Research, The Li Ka Shing Faculty of Medicine, The University of Hong Kong, Hong Kong, China

**Keywords:** Cardiovascular disease, cardiovascular–kidney–metabolic (CKM) syndrome, cirrhosis, hepatocellular carcinoma, metabolic syndrome, mortality

## Abstract

Metabolic dysfunction is a concept derived from metabolic syndrome, which is characterized by the presence of metabolic abnormalities, including obesity, diabetes mellitus, hypertension and dyslipidaemia. Metabolic dysfunction not only drives the development of metabolic dysfunction-associated steatotic liver disease but is also responsible for the heightened cardiovascular risk within the broader scope of liver–cardiovascular–kidney–metabolic syndrome. In other chronic liver diseases, metabolic dysfunction alters the natural history and might impact treatment outcomes.

The global prevalence of metabolic dysfunction-associated steatotic liver disease (MASLD) has increased over the past three decades and is estimated to be 30% as of 2022.^1^ As an indication of disease burden, MASLD-related liver transplantation has already surpassed many other aetiologies as a leading indication of transplant waitlisting in the West.^2^ Metabolic dysfunction underlies the pathogenesis of MASLD and is defined when at least one of the five cardiometabolic conditions, encompassing overweight/obesity, prediabetes/diabetes, hypertension, hypertriglyceridaemia and low high-density lipoprotein cholesterol, is present, which leads to lipid accumulation in the liver, steatohepatitis, cirrhosis and liver cancer.^3^ The concept of metabolic dysfunction emerged from the more well-known and established metabolic syndrome, which requires at least three out of five criteria for diagnosis.^4^ Incorporating metabolic dysfunction into the diagnostic criteria for MASLD aids the identification of patients who have high-risk phenotypes, including significant liver fibrosis and abnormal liver enzymes, as well as high-risk groups for developing adverse hepatic outcomes. This contrasts with the old criteria, ‘non-alcoholic fatty liver disease (NAFLD)’, which is basically a diagnosis by exclusion. Among the disease-defining conditions, type 2 diabetes (T2D) is associated with the highest risk of disease progression in MASLD. In a study of 2,016 participants with MASLD, T2D was associated with a significantly increased risk of hepatic decompensation and hepatocellular carcinoma compared with participants without T2D.^5^ A higher number of cardiometabolic criteria was found to be associated with a higher cumulative incidence of major adverse cardiovascular events, but not liver-related events.^6^ To quantify the severity of metabolic dysfunction, apart from measuring the homeostatic model assessment for insulin resistance, circulating sulphatides – a type of sulphated glycosphingolipids – and highly sensitive C-reactive protein have been shown to correlate with liver fibrosis severity in MASLD.^7,8^ Therefore, metabolic dysfunction is not just a defining feature of MASLD but also contributes to disease prognostication and outcome prediction. MASLD should be actively sought among patients with evidence of metabolic dysfunction frequently encountered in endocrinology or primary care settings, starting with imaging to confirm the presence of hepatic steatosis, followed by non-invasive tests (such as Fibrosis-4 Index [FIB-4] or vibration-controlled transient elastography) to stratify the risk of liver fibrosis.

Apart from MASLD and its disease-defining conditions, many other metabolic diseases, such as chronic kidney disease, heart failure and stroke, share common pathophysiological mechanisms, and it was recently suggested to adopt a new staging structure called cardiovascular–kidney–metabolic (CKM) syndrome, highlighting a multi-system disorder to enhance prevention, risk stratification and multidisciplinary management of these intercalated conditions.^9^ There are five stages that encompass the spectrum of risk and window for intervention: stage 0, no CKM risk factors; stage I, excess or dysfunctional adiposity; stage II, metabolic risk factors; stage III, subclinical cardiovascular disease and stage IV, clinical cardiovascular disease. It is worth highlighting the prominent role of adiposity in CKM syndrome, for the fact that adipose tissue is recognized as an active endocrine organ that contributes to inflammation and metabolic dysfunction.^10^ When considering MASLD in the context of CKM syndrome, more patients with MASLD fell into CKM stage II or III but not stage IV compared with the general population, as shown in a retrospective analysis of the National Health and Nutrition Examination Survey (NHANES) 2017–2020 and NHANES III cohorts.^11^ The prevalence of advanced liver fibrosis defined by FIB-4 >2.67 was 0.00%, 4.35%, 17.28%, 40.24% and 38.12% for CKM stage 0, I, II, III and IV, respectively.^11^ Similar multispecialty practice recommendations for complex conditions involving diabetes, cardiorenal and/or metabolic diseases have been recently updated and expanded for stratified assessments and targeted treatment plans for the ‘hepato-adipo-cardio-renal axis’, and some have even proposed a new term, ‘cardiovascular–renal–hepatic–metabolic’ (CRHM) syndrome.^12,13^ In fact, the liver is recognized for its role in insulin resistance, lipid metabolism, endothelial dysfunction and systemic inflammation, and therefore should be repositioned at the centre of CKM syndrome (i.e. liver–CKM).^13,14^ Emerging research has looked into the pathogenesis of liver–CKM, exemplified by an interesting commentary by Wimalarathne et al., which proposed a novel link between clonal haematopoiesis of indeterminate potential (CHIP), an age-related phenomenon, and driving systemic inflammation, which also increases the risk of metabolic dysfunction-associated steatohepatitis and fibrosis, elucidating the role of CHIP in the progression of liver–CKM syndrome.^15^ Overall, MASLD is a manifestation of a broader clinical syndrome (be it CKM, liver–CKM or CRHM), and treatments targeting the underpinning metabolic dysfunction are needed for reduction of all-cause mortality in patients with MASLD, considering the fact that cardiovascular disease and extrahepatic malignancies remain the leading causes of death among people living with MASLD.^1,16,17^

Beyond MASLD, metabolic dysfunction is frequently seen in patients with other chronic liver conditions. In chronic hepatitis B infection, both body mass index and T2D have been implicated in fibrosis progression, of which adipokines, including adipocyte fatty acid-binding protein and fibroblast growth factor 21, have been implicated.^18–20^ Concomitant hepatic steatosis, as a manifestation of metabolic dysfunction, is observed in 47.8% and is associated with fibrosis progression and hepatitis B surface antigen seroclearance.^21,22^ The number of cardiometabolic risk factors, instead of hepatic steatosis *per se*, was associated with all-cause and liver-specific mortality.^23^ The high prevalence of concomitant chronic hepatitis B infection and MASLD, together with accumulating data on its clinical implications, has sparked interest among some researchers, who proposed a novel entity of metabolic dysfunction superimposed with chronic hepatitis B infection (Met-HBV) that awaits further evaluation.^24^ In patients with primary biliary cholangitis, concomitant MASLD was associated with worse prognosis in terms of inadequate biochemical response to ursodeoxycholic acid treatment by Paris II criteria (61.1% versus 33.3%; p=0.004) and higher liver-related mortality or liver transplantation (22% versus 7.5%; p=0.03).^25^ In patients who have undergone liver transplantation, *de novo* hepatic steatosis was found in one-quarter of patients and was associated with graft dysfunction.^26^ Having concomitant aetiologies of chronic liver disease in MASLD has been shown to increase the risk of extrahepatic cancers and cancer-related mortality in a non-linear manner.^27^ These examples call for more research in patients with all types of chronic liver disease having concomitant metabolic dysfunction and/or hepatic steatosis. For instance, it should be considered to remove the exclusion criteria of concomitant MASLD in clinical trials for chronic hepatitis B infection.

In summary, metabolic dysfunction represents a key pathogenic and prognostic factor in MASLD and other chronic liver diseases. In patients with any evidence of metabolic dysfunction, MASLD should be actively sought using imaging, followed by non-invasive tests for risk stratification. The clinical syndromes incorporating the liver and other metabolic diseases provide a useful framework for healthcare professionals to acknowledge their implications, streamline management strategies and explore new treatment targets.
